# Predicting factors of symptomatic radiation pneumonitis induced by durvalumab following concurrent chemoradiotherapy in locally advanced non-small cell lung cancer

**DOI:** 10.1186/s13014-021-01979-z

**Published:** 2022-01-15

**Authors:** Hiroshi Mayahara, Kazuyuki Uehara, Aya Harada, Keiji Kitatani, Tomonori Yabuuchi, Shuichirou Miyazaki, Takeaki Ishihara, Hiroki Kawaguchi, Hikaru Kubota, Hideaki Okada, Taira Ninomaru, Chihiro Shindo, Akito Hata

**Affiliations:** 1 Department of Radiation Oncology, Kobe Minimally-invasive Cancer Center, 8-5-1, Minatojima-Nakamachi, Chuo-Ku, Kobe, Hyogo 650-0046 Japan; 2 Division of Radiation Oncology, Kobe University Graduate School of Medicine, 7-5-2, Kusunoki-Cho, Chuo-Ku, Kobe, Hyogo Japan; 3 Department of Respiratory Medical Oncology, Kobe Minimally-invasive Cancer Center, 8-5-1, Minatojima-Nakamachi, Chuo-Ku, Kobe, Hyogo Japan; 4 Department of Diagnostic Radiology, Kobe Minimally-invasive Cancer Center, 8-5-1, Minatojima-Nakamachi, Chuo-Ku, Kobe, Hyogo Japan

**Keywords:** Locally advanced non-small cell lung cancer, Concurrent chemoradiotherapy, Radiation pneumonitis, Dosimetric factor, Durvalumab

## Abstract

**Background:**

Concurrent chemoradiotherapy (CCRT) followed by durvalumab is the standard of care for unresectable locally-advanced non-small cell carcinoma (LA-NSCLC). However, a major concern about administration of durvalumab after CCRT is whether the incidence of symptomatic radiation pneumonitis (RP) may increase or not. In the present analysis, we report the initial results of CCRT followed by durvalumab in patients with LA-NSCLC in a real-world setting with focus on predicting factors for symptomatic RP.

**Methods:**

Patients who were pathologically diagnosed as NSCLC and initiated treatment with CCRT followed by durvalumab between July 2018 to December 2019 were eligible for this study. Patients were included if they completed the planned CRT course and administered at least one course of durvalumab. We retrospectively investigated the preliminary survival outcome and incidence and predicting factors for symptomatic RP.

**Results:**

Of the 67 patients who planned CCRT, 63 patients completed the entire CCRT course. Of these, 56 patients proceeded to consolidation with durvalumab. The median time to eternal discontinuation of durvalumab was 9.7 months. The cumulative proportion of the patients who exhibited symptomatic RP was 30, 40 and 44% at 3, 6 and 12 months, respectively. In multivariate analyses, pulmonary fibrosis score and lung V40 were significant predictive factors for symptomatic RP (*p* < 0.001, HR: 7.83, 95% CI: 3.38–18.13, and *p* = 0.034, HR: 3.17, 95% CI: 1.09–9.19, respectively).

**Conclusions:**

Pulmonary fibrosis sore and lung V40 were significant predictive factors for symptomatic RP. We should be cautious about the administration of durvalumab for patients having subclinical pulmonary fibrosis. To our best knowledge, this is one of the first report showing the predictive value of high dose volumes to the lung in patients with LA-NSCLC who received CCRT followed by durvalumab.

**Supplementary Information:**

The online version contains supplementary material available at 10.1186/s13014-021-01979-z.

## Introduction

Lung cancer is the most frequently diagnosed cancer and is the leading cause of cancer mortality, world widely [1]. Locally advanced Stage 3 non-small cell lung cancer (LA-NSCLC) accounts for 20% of lung cancer cases [2]. Because of frequency in both of locoregional and distant recurrences, concurrent chemoradiotherapy (CCRT) has long been a standard of care for decades [3, 4]. The 5-year overall survival (OS) ratio has estimated to be only 15-30% [5–10]. Numerous studies had tested combination of new systemic agents or dose escalation and failed to improve outcomes [6, 8, 9, 11, 12]. Several studies investigated consolidative chemotherapy after CCRT and showed no apparent clinical benefit [13–17].

The PACIFIC phase 3 randomized controlled trial demonstrated efficacy of consolidation therapy with durvalumab [18–20]. Durvalumab is a selective human IgG1 monoclonal antibody that blocks programmed death ligand-1(PD-L1) binding to PD-1 receptor and CD80, and it increases the anti-tumor activity by T cells [21–23]. In the PACIFIC study, for patients with LA-NSCLC, durvalumab administered after CCRT improved median Progression-free survival (PFS) by 17.2 months compared to its placebo of 5.6 months. The median OS was 47.5 months with durvalumab but was 29.1 months with placebo [20]. Now, administration of durvalumab after CCRT has become to be a standard of care [24].

A major concern about administration of durvalumab after CCRT is whether the incidence and severity of radiation pneumonitis (RP) may increase or not. In the PACIFIC study, RP was observed in 34 % and 25% of the patients with durvalumab and placebo, respectively [18]. In particular, grade 3 and 4 RP occurred in 3.4 % and 2.6 % of patients with durvalumab and placebo, respectively. In the PACIFIC study, patients were randomly assigned to groups after the successful completion of CCRT and those who exhibited symptomatic RP during and immediately after the CCRT were excluded from study inclusion [18]. The reported incidence of RP in the PACIFIC study may not represent a real-world incidence, because it might include only well-conditioned patients. Additionally, actual dosimetric factors, such as lung dose, target coverage, irradiation techniques, or quality of radiotherapy plans were not evaluated, because the part of CCRT was not included in the protocol of the PACIFIC study [18].

In the present analysis, we report the results of CCRT followed by durvalumab in patients with unresectable LA-NSCLC in a real-world setting with focus on predicting factors for symptomatic RP.

## Materials and methods

### Study subjects

Patients with either unresectable primary LA-NSCLC and locoregional recurrent NSCLC after primary resection were included in this study. Patients who were pathologically diagnosed as NSCLC and initiated treatment with CCRT followed by durvalumab between July 2018 to December 2019 were eligible for this study. The data cut-off date was August 31, 2020.

### Patient characteristics

Fifteen-six patients with LA-NSCLC who completed CCRT and received maintenance therapy with durvalumab were eligible for this analysis. Patients’ baseline characteristics are summarized in Table 1. Between July 2018 and December 2019, a total of 78 patients received definitive radiotherapy in our single institution. Among them, 63 were with unresectable primary LA-NSCLC and 15 were with unresectable locoregional recurrent NSCLC after primary resection. Excluding 12 patients who were planned to be treated with radiotherapy alone, 67 patients were planned to receive CCRT. Sixty-three patients completed planned CCRT course, whereas 4 patients discontinued CCRT because of massive respiratory bleeding, tracheoesophageal fistula, chemotherapy-induced pneumonitis, and patient’s refusal for chemotherapy, respectively. Excluding these 4 patients, 63 patients completed CCRT. Of these, 56 patients received durvalumab after a median of 19 days from the last day of irradiation. Seven patients did not receive durvalumab, due to surgical resection in 2, comorbidity in 2, early symptomatic RP in 1, deteriorated performance status in 1 and patient’s refusal in 1, respectively. These patients were excluded from further analysis to maintain comparability with the results of PACIFIC study. Thus, 56 of 67 (84%) patients who planned CCRT proceeded to maintenance therapy with durvalumab. Applied irradiation techniques were intensity modulated radiotherapy (IMRT) for 28 patients and 3D-conformal radiation therapy (3D-CRT) for 28 patients.

**Table 1:** Patients' characteristics

Characteristics	N = 56 (%)
Age	
Median years (range)	72 (48–85)
Gender	
Male/female	37 (66) / 19 (34)
Performance status	
0 / 1 / 2	24(43) / 28 (50) / 4 (7)
Smoking status	
Current / Former / Never	20(36) / 25(45) / 11 (20)
Histology	
Adeno / Sq / Non-small	25(45) / 30(54) / 1(2)
Primary tumor location	
Upper lobe or trachea / Middle or lower lobe	33(59) / 23 (41)
Clinical stage	
|||A/|||B/|||C/Others	19(34) /14(25) /10(18) /13 (23)
PD-L1 Satus	
≥ 50% /1–49% / < 1% / unknown	9(16) /11(20) / 19(24) / 17(30)
Irradiation technique	
IMRT	28 (50)
3D-CRT	28 (50)
Total radiotherapy dose	
60 Gy/30fr	48 (86)
66 Gy/33fr	5 (9)
54 Gy/27fr	1 (2)
50 Gy/25fr	2 (4)
Chemotherapy regimen	
wCBDCA + PTX	26 (46)
CDDP + VNR	12 (21)
CDDP + Pemetrexed	12 (21)
CDDP + S-1	6 (11)
Pulmonary function test	
Median %VC (range)	89.0 (53.5–124.4)
Median %FEV1.0 (range)	80.6 (46–126)

*Adeno* adenocarcinoma, *Sq* squamous cell carcinoma, *PD-L1* programmes cell death -ligand 1, *IMRT* intensity modulated radiation therapy, *3D-CRT* three dimensional-conformal radiation therapy, *fr* fractions, *w* weeekly, *CBDCA* carboplatin, *PTX* paclitaxel, *CDDP* cisplatin, *VNR* vinorelbine, *VC* vital capacity, *FEV* forced expiratory volume

The patient’s consent for the treatment was obtained in a written form. Clinical staging was done by fluorodeoxyglucose-positron emission tomography, contrast-enhanced computed tomography (CT) and gadolinium-enhanced magnetic resonance imaging (MRI) of the brain, according to the Union for International Cancer Control criteria (8th ed.). Patients were included if they completed the planned CRT course and administered at least one course of durvalumab. We retrospectively investigated the incidence and predicting factors for symptomatic RP. This study was approved by our Institutional Review Board and was conducted in accordance with the Declaration of Helsinki.

### Statistical analysis

The primary objective of this analysis was to describe the clinical outcomes associated with CCRT followed by durvalumab. OS and PFS was estimated as the time from starting CCRT to death or disease progression, by using the Kaplan-Meier method.

Possible clinical and dosimetric factors that may predict symptomatic RP were statistically investigated. Symptomatic RP was defined as Grade 2 or higher RP (G2RP) by the Common Toxicity Criteria for Adverse Events (Version 5.0). The time to G2RP was defined as the time from completion of CCRT to the development of G2RP and was calculated by using a Kaplan–Meier estimator, and compared by using a log-rank test. Time to discontinue durvalumab (TTDD) was defined as the time from the first administration of durvalumab to 14 days after the last administration of durvalumab. Temporary postponement of durvalumab due to toxicity, or completion after 12 month of administration was not counted for an event. Disease progression and discontinuation of durvalumab by the reason other than RP were treated as competing risk for TTDD due to RP, and the hazard ratio (HR) was estimated using the Fine-Gray method.

The percent of lung volumes receiving above various dose levels were statistically evaluated. The parameters assessed included percentage of total lung volume (lung minus gross tumor volume) exceeding 50Gy (V50), 40Gy (V40), 30Gy (V30), 20Gy(V20), 10Gy (V10), 5Gy (V5), mean lung dose (MLD), volume of the lung received less than 5 Gy (Vs5) and initial planning target volume (PTV). For detecting optimal cut-off values of continuous variables, we underwent receiver-operating characteristic (ROC) analyses, and the optimal cut-off values were determined by Youden index. Then, areas under the curve (AUC) were calculated for each value. Associations between dosimetric variables were evaluated by using the Pearson correlation coefficient. A correlation coefficient of more than 0.6 was regarded as having some correlation between variables. When we faced with factors that were correlated with each other, we selected the factor that had the highest area under the curve (AUC) in ROC analyses. Multivariate analyses by using Fine-Gray model were performed including factors that had shown significant associations (*p* < 0.05) in univariate Gray’s test.

All analyses were performed in R, version 3.6.3 (R Foundation for Statistical Computing). All hypothesis tests were 2-sided and a *p* < .05 was considered statistically significant.

### Image analysis

In regard to the evaluation of baseline lung fibrosis, we used pulmonary fibrosis score, which was declared by Kazerooni EA, et al and modified by Tsujino et al. [25, 26]. Pulmonary fibrosis was scored according to the extent of the subpleural focal honeycombing. The scoring definition of pulmonary fibrosis is shown in Table 2. Pulmonary fibrosis scores were independently reviewed by an experienced diagnostic radiologist, pulmonary medical oncologist and radiation oncologist, those who were blinded from patient’s medical records. If there was any discordance in an evaluation in pulmonary fibrosis score, the score was decided on discussion among them. Interreader agreement analysis was not performed.

**Table 2:** Definition of pulmonary fibrosis score

Score	Definition
0	No fibrosis
1	Interlobular septal thickening; no descrete honeycoming
2	Honeycoming (with or without septal thickening) involving < 25% of the lobe
3	Honeycoming involving 25–49% of the lobe
4	Honeycoming involving 50–75% of the lobe
5	Honeycoming involving > 75 of the lobe

### Radiotherapy

Radiotherapy was delivered using a 10 or 6-MV Xray by TrueBeam (Varian Medical Systems, CA, USA). Four-dimensional CT (4-DCT) was used to evaluate respiratory tumor motion. Varian’s RPM respiratory-gating irradiation system was used if the respiratory tumor motion encompasses 10mm. For dose calculation, images of expiratory phase (a 2 mm thickness) were used. The Eclipse (ARIA 11.0.42, Varian Medical Systems, CA, USA) treatment planning software was used for dose optimization and calculation. Irradiation techniques included both of IMRT and 3D-CRT. The irradiation technique was decided at the discretion of the attending radiation oncologist, in consideration of the anatomical tumor location, tumor extension and treatment schedule. All the irradiations were delivered under image guidance by orthogonal on-board imager (OBI) and kV cone beam CT (CBCT). Gross target volume (GTV) of the primary lesion was defined in simulated CT images of the lung window. Internal target volume (ITV) was determined by the summation of GTVs in 4-D CT images to encompass whole respiratory tumor motion. In case of respiratory-gating, ITV was determined as summation of GTVs in only end-respiratory phase (typically, 40-60% of the respiratory cycle). Clinical target volume (CTV) included a 5 mm margin in all directions from ITV. Prophylactic regional irradiation was basically not applied. A PTV was defined as CTV with a 4–5 mm margin to compensate for any set-up error. Prescribed dose was 60 Gy in 30 fractions for all the patients, except for one case who discontinued irradiation at a dose of 54 Gy in 27 fractions, due to infectious pneumonitis. The dose was prescribed to an isocenter in a case for 3D-CRT, whereas the dose was prescribed to D50% of the PTV in a case for IMRT until April 2019. Then it was switched to D95% of the PTV thereafter, in accordance with protocol of another prospective observational clinical study. Dose constraints for organs at risk were <45 Gy to spinal cord and V20, V5 of the lung should be < 30%, < 65%, respectively.

### Chemotherapy

The concurrent chemotherapy regimens included weekly carboplatin + paclitaxel (PTX), cisplatin (CDDP) +S-1, CDDP + vinorelbine and CDDP + pemetrexed. The regimen was determined at the discretion of the attending medical oncologists depending on the patients’ age, general condition, organ functions and tumor histology.

### Durvalumab

Diagnostic CTs were taken immediately after completing CCRT to evaluate its efficacy and to detect RP. If no abnormalities were found on CT and blood tests, durvalumab was started. Durvalumab (10 mg/kg) was administered intravenously every 2 weeks until 1 year [18]. The administration of durvalumab was continued until disease progression, emergence of unacceptable toxicities such as G2RP or withdrawal of consent. If patients developed G2RP, they typically were treated by corticosteroids with prednisolone of 0.5-1.0mg/kg, and the administration of durvalumab were postponed until they resolved the symptom and reduced prednisolone to a dose of less than 5-10mg per body.

### Follow-up

After starting durvalumab, patients were suggested to receive chest X-ray and blood test for every bi-weekly visit for durvalumab. Chest and upper abdominal CT images were taken for every 2 months for the first year, every 3–4 months thereafter. Brain MRI were taken for every 6 months.

## Results

### OS, PFS and cause of morbidity

**Fig. 1:**
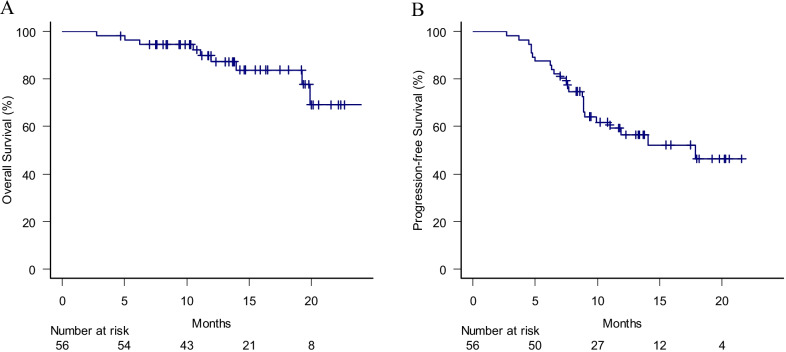
**a** Kaplan–Meier curve for overall survival of the eligible patients. **b** Kaplan–Meier curve for progression-free survival of the eligible patients

With a median follow-up period of 14.0 months for the living patients, the 12- and 18-months OS ratio were 87 and 84%, respectively (Fig. 1a). At the time of analysis, 9 patients had deceased. Six of them had died from primary disease progression, 1 from another cancer and 2 from treatment-related toxicities (lung toxicity in one and toxic epidermal necrolysis in one). The 12- and 18-months PFS were 57 and 46%, respectively (Fig. 1b).

### Continuity of durvalumab

**Fig. 2:**
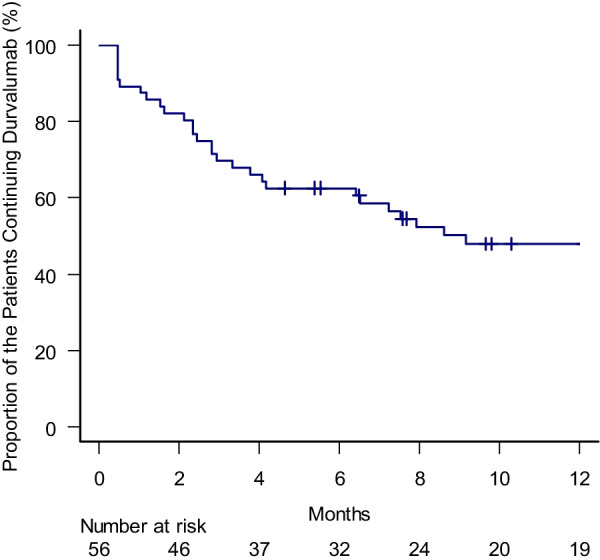
Kaplan–Meier curve for proportion of the patients who were continuing durvalumab

At the time of analysis, 19 patients completed 1 year of durvalumab administration, whereas 8 patients were currently under administration. Twenty-nine patients discontinued durvalumab. Of these, 15 discontinued durvalumab due to disease-progression, 11 by toxicity, and 3 from patient’s refusal. The proportion of the patients who were continuing durvalumab at 3, 6 and 12 months was 70, 63 and 48%, respectively (Fig. 2). The median TTDD was 9.7 months.

### Incidence of radiation pneumonitis

**Fig. 3:**
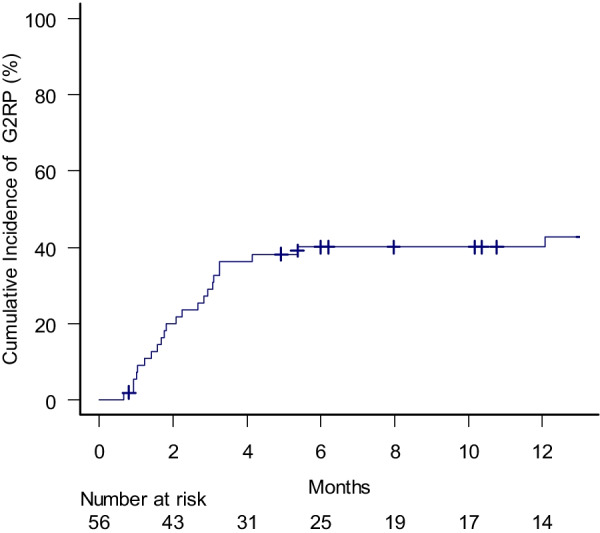
Cumulative incidence of radiation pneumonitis of grade 2 or more

**Fig. 4:**
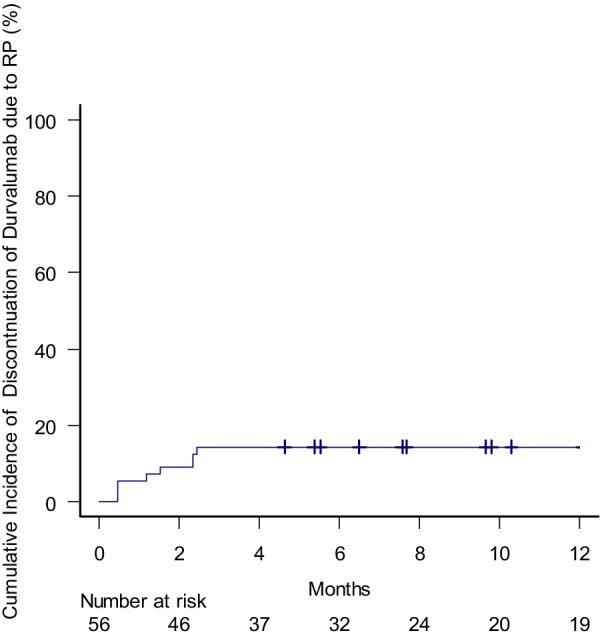
Cumulative incidence of the patients who eternally discontinued durvalumab due to radiation pneumonitis

The number of the patients who developed RP of Grade 0, 1, 2, 3 and 5 were 6 (10.7%), 28 (50%), 17 (30.4%), 4 (7.1%) and 1(1.8%), respectively. Case presentations on the typical clinical courses of radiation pneumonitis are available in the Additional file 1: Appendix 1 and 2. The cumulative proportion of the patients who exhibited G2RP was 30, 40 and 44% at 3, 6 and 12 months, respectively (Fig. 3). Oral prednisolone of 0.5–1.0mg/kg was administered to 19 out of the 22 patients with G2RP. Six patients resumed durvalumab after the remission of RP. The proportion of the patients who eternally discontinued durvalumab due to G2RP was 14, 14 and 14% at 3, 6 and 12 months, respectively (Fig. 4).

### ROC analysis of the dose-volume histogram parameters of the lung for G2RP

The results of ROC analysis for G2RP are summarized in Table 3. The patients were dichotomized according to the threshold levels determined by the ROC analysis. Cumulative incidences of G2RP were estimated by the Gray’s test. Discontinuation of durvalumab due to the cause other than G2RP was treated as a competing risk for G2RP. Lung V30, V40, V50, mean lung dose (MLD) and initial planning target volume (PTV) were significant predictors for G2RP. Lung V20, V10 and V5 did not significantly predicted G2RP. Pearson correlation coefficients between Lung V30/V40, V40/V50 and V30/V50 were 0.730, 0.853, and 0.629, respectively. Pearson correlation coefficients between MLD/V30, MLD/V40 and MLD/V50 were 0.762, 0.802, and 0.661, respectively. Because lung V30, V40, V50 and MLD were correlated with each other, we selected lung V40, which had highest AUC among them, for further analysis.

**Table 3:** ROC analysis of the dose-volume histogram parameters of the lung and incidence of radiation pneumonitis of grade 2 or more

Dose (Gy)	AUC	Threshold level (%)	Cumulative incidence of G2RP at 6 months		*p* value
			≥ threshold	< threshold	
Lung V50	0.640	5.3	56.8	13.6	0.008
Lung V40	0.686	10.0	57.3	16.7	0.011
Lung V30	0.644	15.7	57.3	26.1	0.048
Lung V20	0.608	23.0	56.4	29.8	0.16
Lung V10	0.566	34.1	52.9	30.0	0.12
Lung V5	0.570	48.2	55.0	30.5	0.07
Mean lung dose	0.640	12.1	54.0	21.1	0.046
Lung Vs5 (ml)	0.533	1364	41.1	37.6	0.62
Initial PTV (ml)	0.679	398	58.3	23.8	0.024

*ROC* receiver-operating characteristic, *G2RP* radiation pneumonitis of grade2 or more, *Lung Vx* percentage of the lung volume exceeding x Gy, *Lung Vs5* volume of the lung received less than 5 Gy, *PTV* planning target volume

### Univariate and multivariate analyses of factors affecting risk of G2RP

**Fig. 5:**
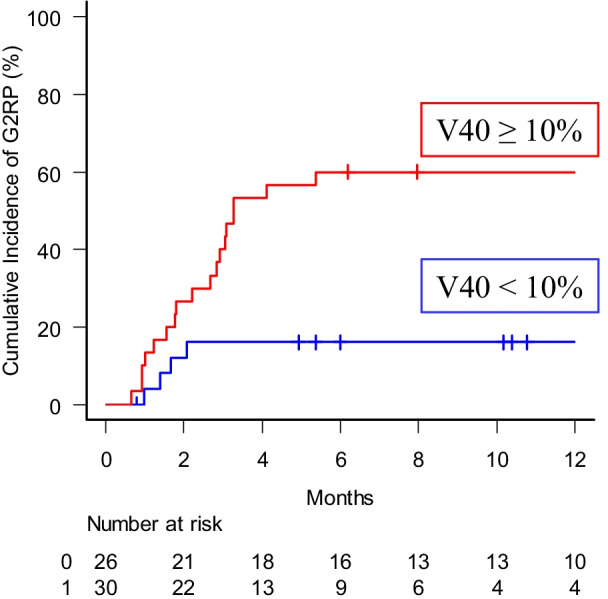
Cumulative incidence of radiation pneumonitis of grade 2 or more stratified by lung V40. The dichotomizing value was based on ROC analysis. Lung V40: percentage of lung volume exceeding 40 Gy

Univariate analyses for G2RP included age, gender, performance status, pulmonary fibrosis score, pulmonary function test, smoking history, primary tumor location, clinical stage, concurrent chemotherapeutic regimen, irradiation technique, lung V40, and initial PTV. Univariate analyses revealed that gender (male), pulmonary fibrosis score (≥ 2), smoking history (present), lung V40 (≥ 10%) and initial PTV (≥ 398ml) were significant predictor for G2RP (Table 4). There was no difference in the incidence of G2RP between IMRT and 3DCRT. Pulmonary function was also not a predictive factor for G2RP. The variables that showed significance in the univariate analyses were further evaluated in multivariate analyses. In multivariate analyses, pulmonary fibrosis score and lung V40 remained to be significant factors for G2RP (*p* < 0.001, HR: 7.83, 95%CI: 3.38–18.13, and *p* = 0.034, HR: 3.17, 95% CI: 1.09–9.19, respectively). The cumulative incidence of G2RP at 6 months was 16.7% and 57.3% with lung V40 of below and above the threshold level of 10%, respectively (Fig. 5).

**Table 4:** Univariate and multivariate analyses of factors affecting risk of radiation pneumonitis of Grade 2 or more and discontinuation of durvalumab Univariate analysis

Variables		N	6 M cumulative incidence of G2RP (%)	*p* value	6 M cumulative incidence of discontinuation of Durvalumab due to G2RP (%)	*p* value	
Gender	Male	37	48.7	0.038	21.6	0.03	
	Female	19	18.6		0.0		
Pulmonary Fibrosis Score	≥ 2	10	90.0	< 0.001	60.0	< 0.001	
	0–1	46	28.3		4.3		
Smoking history	Present	45	46.5	0.031	17.8	0.14	
	Never	11	9.0		0.0		
Lung V40	≥ 10%	30	57.3	0.011	20.0	0.21	
	< 10%	26	16.7		7.7		
Initial PTV	≥ 398 ml	24	58.3	0.024	25.0	0.048	
	< 398 ml	32	23.8		6.3		

Multivariate analysis							
Variables		HR	95% CI	*p* value	HR	95% CI	*p* value
Pulmonary Fibrosis Score	≥ 2	7.83	3.38–18.13	< 0.001	5.89	1.53–22.68	< 0.001
Lung V40	≥ 10%	3.17	1.09–9.19	0.034			
Initial PTV					2.62	0.71–9.71	0.15

*Lung V40* percentage of the lung volume exceeding x Gy, *Lung Vs5* volume of the lung received less than 5 Gy, *HR* hazard ratio, *PTV* planning target volume

### Univariate and multivariate analyses of factors affecting risk of eternal discontinuation of durvalumab due to RP

The variables showed some significance for predicting G2RP were included in the univariate analyses. Univariate analyses revealed that gender (male), pulmonary fibrosis score (≥ 2) and initial PTV (≥398ml) were significant factors for eternal discontinuation of durvalumab (Table 4). There was also no difference in the incidence between IMRT and 3DCRT. The variables that showed significance in the univariate analyses were further evaluated in multivariate analysis. In multivariate analysis, only pulmonary fibrosis score remained to be a significant factor (< 0.001, HR: 5.89, 95% CI: 1.53–22.68).

### Cumulative incidence of G2RP according to lung V20 level

**Fig. 6:**
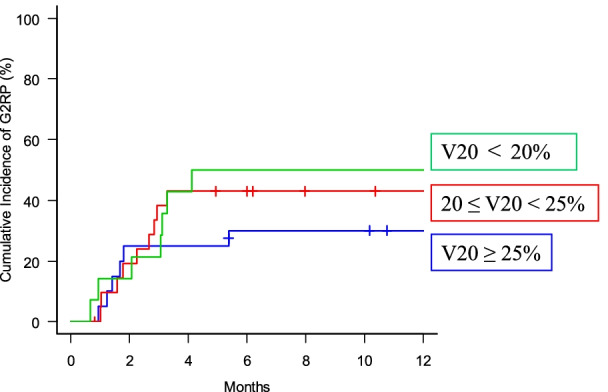
Cumulative incidence of radiation pneumonitis of grade 2 or more according to lung V20 level. Lung V20: percentage of lung volume exceeding 20 Gy

The 6 months-cumulative incidence of G2RP among patients with lung V20 of <20%, 20–25% and V20≥25% were 25.0, 46.7 and 51.8% respectively (Fig. 6). There were no statistical differences among them (*p* = 0.51).

## Discussion

In the current study, the incidence of G2RP was 39.3% after CCRT followed by durvalumab for LA-NSCLC. The incidence seems to be higher than the previous reports without durvalumab [26–29]. From recent multi-institutional retrospective analysis in Japan, the incidence of 24% for G2RP were reported before introduction of durvalumab [29]. Few reports had reported the real-world incidence of G2RP when durvalumab is administered after CCRT. In coincidence with the current study, reports from several institutions revealed the incidence to be 36–43% [30–32]. Jung et al reported the higher incidence of G2RP among patients administered durvalumab, compared to observed patients (42.9% vs. 20%) [31]. They also reported the higher incidence of Grade 3 RP in the patients with durvalumab, compared to observation (14.3% vs. 2.5%). Recently reported multi-institutional study in Japan revealed that the incidence of G2RP were 37.7% with durvalumab [32]. Consolidation with durvalumab should increase the incidence of symptomatic RP, especially in Asian ethnicity patients.

In the consideration for durvalumab, development of G2RP is a clinically important endpoint. One of frequent reasons for discontinuing durvalumab is symptomatic RP. When a patient develops G2RP, durvalumab is interrupted and typically be treated by corticosteroid therapy. Interruption of durvalumab as well as immunologic inhibition by corticosteroid may impair the anti-tumor activity by T cells, which has been enhanced by durvalumab. Therefore, prediction and prevention of G2RP is crucial.

Dosimetric analysis of this study showed that the percentage of the lung irradiated exceeding 40Gy (V40) were independent predictors for G2RP. Various predicting factors for RP were reported so far [27, 28, 33–35]. Among them, lung doses have been regarded as the most distinct predicting factor for RP. In 2003, Tsujino et al. reported the relationships between lung V20 and the incidence of G2RP in CCRT for LA-NSCLC [28]. The lung V20 of higher than 25% significantly increased the incidence of G2RP [28]. To reduce the incidence of RP, introduction of new irradiation techniques, such as IMRT or respiratory motion management would be useful [36–38]. However, there is another concern about the risk of excessive low dose irradiation to the lung from the reports of post extrapleural pneumonectomy radiotherapy for pleural mesothelioma [39, 40]. In the case with CCRT using IMRT for LA-NSCLC, the incidence of G3 RP significantly increased when lung V5 exceeded more than 70% [41]. In this study, there was no difference in the incidence of Grade 2 or 3 RP between patients who received IMRT or 3D-CRT, and neither V20 nor V5 was significant predictor for G2RP. In contrast to previous reports, in the current study, the volume of the lung irradiated to high dose (V40) found to be independent significant predictors of G2RP. Some previous reports declared the high dose constraint, however, it had not often been highlighted in recent reports [42, 43]. Both of lung V20 and V5 were strictly restricted in the treatment planning in our general practice, irrespective of the irradiation techniques. On the other hands, we did not restrict lung V30 to 50. Possible reason for the correlation between lung V40 and RP in our cohort might be that the variations in lung V40 were larger than that of V20 and V5. Recently, in line with the current study, Saito et al suggested significant association of medium to high dose-volumes of the lung and G2RP in patients with LA-NSCLC treated with CCRT followed by durvalumab [30]. High dose volumes to the lung should also be associated with the incidence of G2RP in patients with LA-NSCLC treated with CCRT followed by durvalumab. Every effort should be practiced to reduce the high dose irradiated volume of the lung.

In the current study, baseline existence of pulmonary fibrosis was the strongest predictor of G2RP and only an independent predictor of permanent discontinuation of durvalumab due to RP. Association between subclinical interstitial lung disease and fatal radiation pneumonitis was described in several reports [44–46]. Tsujino et al advocated the predictive risk score including subclinical interstitial lung disease for Grade 3 RP [26]. Pulmonary fibrosis score of 2 or more, which has honeycoming, was an independent predictor for Grade 3 RP. When pulmonary fibrosis was scored in combination with another predictors (age≥68, lung V20≥26% and lung Vs5 <1500cc), the predictability for Grade 3 RP was significantly improved. Taking into consideration of this predictive risk sore in the treatment planning for LA-NSCLC, the incidences of Grade 3 or higher RP radically reduced over time in their institution (personal communication). Careful patient selection for durvalumab is crucial especially for patients who are suspected to have subclinical interstitial lung disease.

Preliminary results of OS and PFS of the current study seems to be comparable to the initial report of the PACIFIC study [18]. However, there were non-negligible difference in the baseline characteristics of the included patients exists between the PACIFC study and the current study. Our study included relatively older patients, with median age of 72, compared to 64 in the PACIFIC study. Additionally, more unfavorable patients, 10 patients (18%) with clinical stage IIIC were included in this study, who were not included in the PACIFIC study. These difference in the patient’s background might increase the incidents of RP. Regardless of the considerable patient selection biases, preliminary survival outcomes of the current study were similar to that of the PACIFIC study. The results of the current study suggested the reproduced survival benefit of durvalumab in a real-world settings.

We know there are several limitations in the current study. Firstly, because of the retrospective nature, patient selection criteria for both of CCRT and durvalumab may vary among attending physicians. Also, the grading of RP which were based on the medical records may have an impact on the interpretation of the results. Secondly, because irradiation technique was determined at the discretion of the attending radiation oncologists, baseline characteristics of the patients who received CCRT with IMRT or 3D-CRT were not matched with each other. Thirdly, the optimal cut-off value of the lung dose-volume still needs to be investigated because of the limitation in the patient number included in the current study. Lastly, possible biomarkers that may predict the incidence or severity of RP were not investigated in the current study, although a part of the patients’ serums were sequentially cryopreserved for future assays under obtained informed consent. We also conducted a multi-institutional prospective clinical trial, WJOG12019L (UMIN000038366) and is currently ongoing to investigate efficacy and safety of CCRT using IMRT followed by durvalumab for LA-NSCLC.

## Conclusions

Pulmonary fibrosis sore and lung V40 were significant predictive factors for symptomatic RP in patients with LA-NSCLC after CCRT followed by durvalumab. We should be cautious about the administration of durvalumab for patients having subclinical pulmonary fibrosis. To our best knowledge, this is one of the first report showing the predictive value of high dose volumes to the lung in patients with LA-NSCLC who received CCRT followed by durvalumab.

## Supplementary Information

**Additional file 1**. **Appendix 1:** A case presentation on typical clinical course of radiation pneumonitis in a patient with high lung V40 value. **Appendix 2:** A case presentation on typical clinical course of radiation pneumonitis in a patient with subclinical lung fibrosis.

## Data Availability

Research data are stored in an institutional repository and will be shared upon request to the corresponding author.

## Abbreviations

CCRT: Concurrent chemoradiotherapy
LA-NSCLC: Locally-advanced non-small cell carcinoma
RP: Radiation pneumonitis
Vx: Percentage of total lung volume exceeding x Gy
HR: Hazard ratio
CI: Confidence interval
OS: Overall survival
IgG: Immunoglobulin G
PD-L1: Programmed death ligand-1
PD-1: Programmed cell death-1
CD80: Cluster of differentiation 80
PFS: Progression-free survival
IMRT: Intensity-modulated radiation therapy
3D-CRT: 3D-conformal radiation therapy
CT: Computed tomography
MRI: Magnetic resonance imaging
G2RP: Grade 2 or higher radiation pneumonitis
TTDD: Time to discontinue durvalumab
MLD: Mean lung dose
Vs5: Volume of the lung received less than 5 Gy
PTV: Planning target volume
ROC: Receiver-operating characteristic
AUC: Areas under the curve
4-DCT: Four-dimensional computed tomography
OBI: On-board imager
CBCT: Cone beam computed tomography
GTV: Gross target volume
ITV: Internal target volume
CTV: Clinical target volume
PTX: Paclitaxel
CDDP: Cisplatin
WJOG: West Japan Oncology Group
