# Supplementary material for: Predicting factors of symptomatic radiation pneumonitis induced by durvalumab following concurrent chemoradiotherapy in locally advanced non-small cell lung cancer
Source: Radiat Oncol. 2022 Jan 15;17:7. doi: 10.1186/s13014-021-01979-z (PMC8760798; doi:10.1186/s13014-021-01979-z)
Supplement: Supplementary file 1 — Additional file 1. Appendix 1: A case presentation on typical clinical course of radiation pneumonitis in a patient with high lung V40 value. Appendix 2: A case presentation on typical clinical course of radiation pneumonitis in a patient with subclinical lung fibrosis. [file 13014_2021_1979_MOESM1_ESM.pptx]

## Slide 1
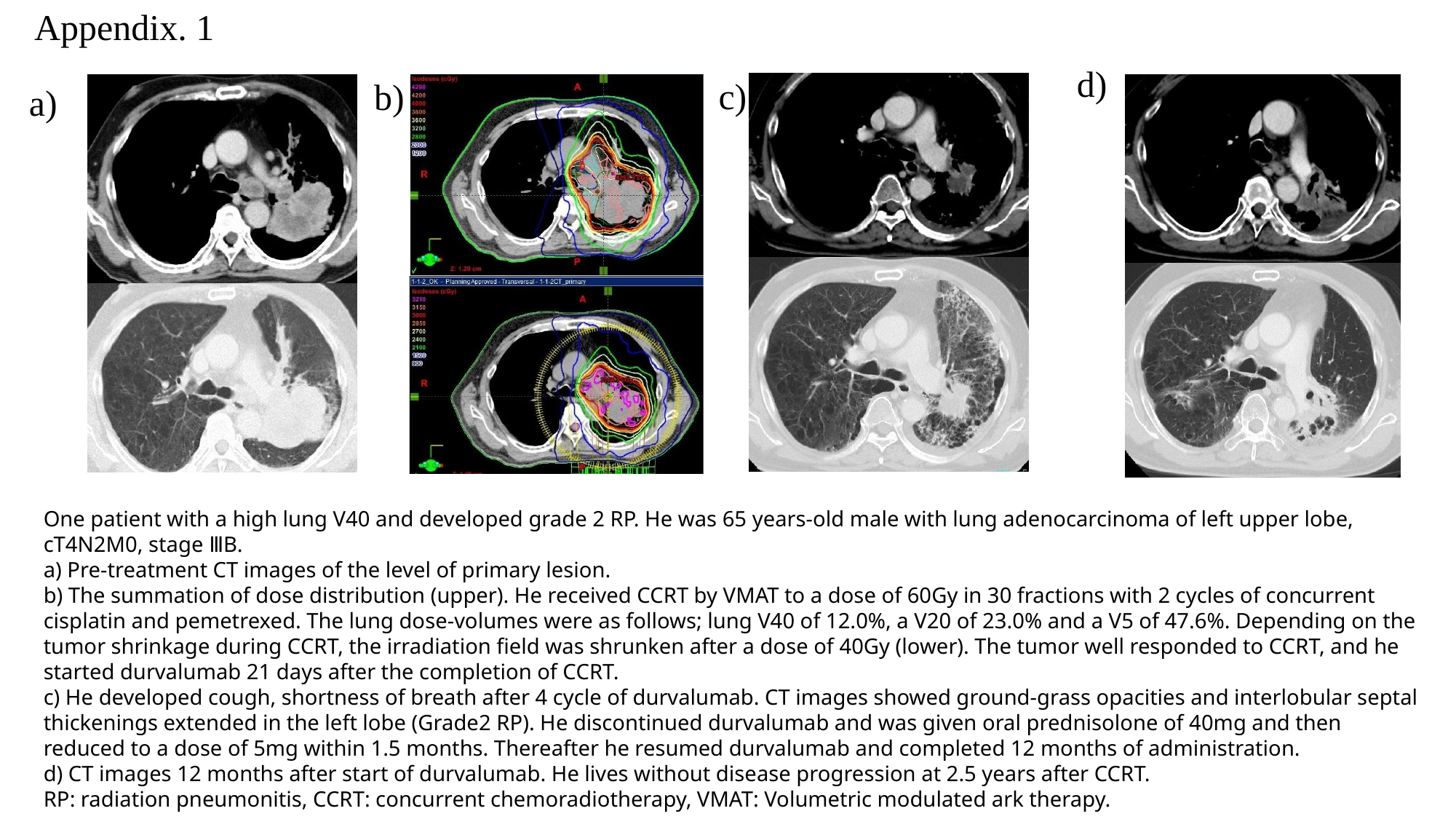

Appendix. 1
d)
c)
b)
a)
One patient with a high lung V40 and developed grade 2 RP. He was 65 years-old male with lung adenocarcinoma of left upper lobe, cT4N2M0, stage ⅢB.
a) Pre-treatment CT images of the level of primary lesion.
b) The summation of dose distribution (upper). He received CCRT by VMAT to a dose of 60Gy in 30 fractions with 2 cycles of concurrent cisplatin and pemetrexed. The lung dose-volumes were as follows; lung V40 of 12.0%, a V20 of 23.0% and a V5 of 47.6%. Depending on the tumor shrinkage during CCRT, the irradiation field was shrunken after a dose of 40Gy (lower). The tumor well responded to CCRT, and he started durvalumab 21 days after the completion of CCRT.
c) He developed cough, shortness of breath after 4 cycle of durvalumab. CT images showed ground-grass opacities and interlobular septal thickenings extended in the left lobe (Grade2 RP). He discontinued durvalumab and was given oral prednisolone of 40mg and then reduced to a dose of 5mg within 1.5 months. Thereafter he resumed durvalumab and completed 12 months of administration.
d) CT images 12 months after start of durvalumab. He lives without disease progression at 2.5 years after CCRT.
RP: radiation pneumonitis, CCRT: concurrent chemoradiotherapy, VMAT: Volumetric modulated ark therapy.

## Slide 2
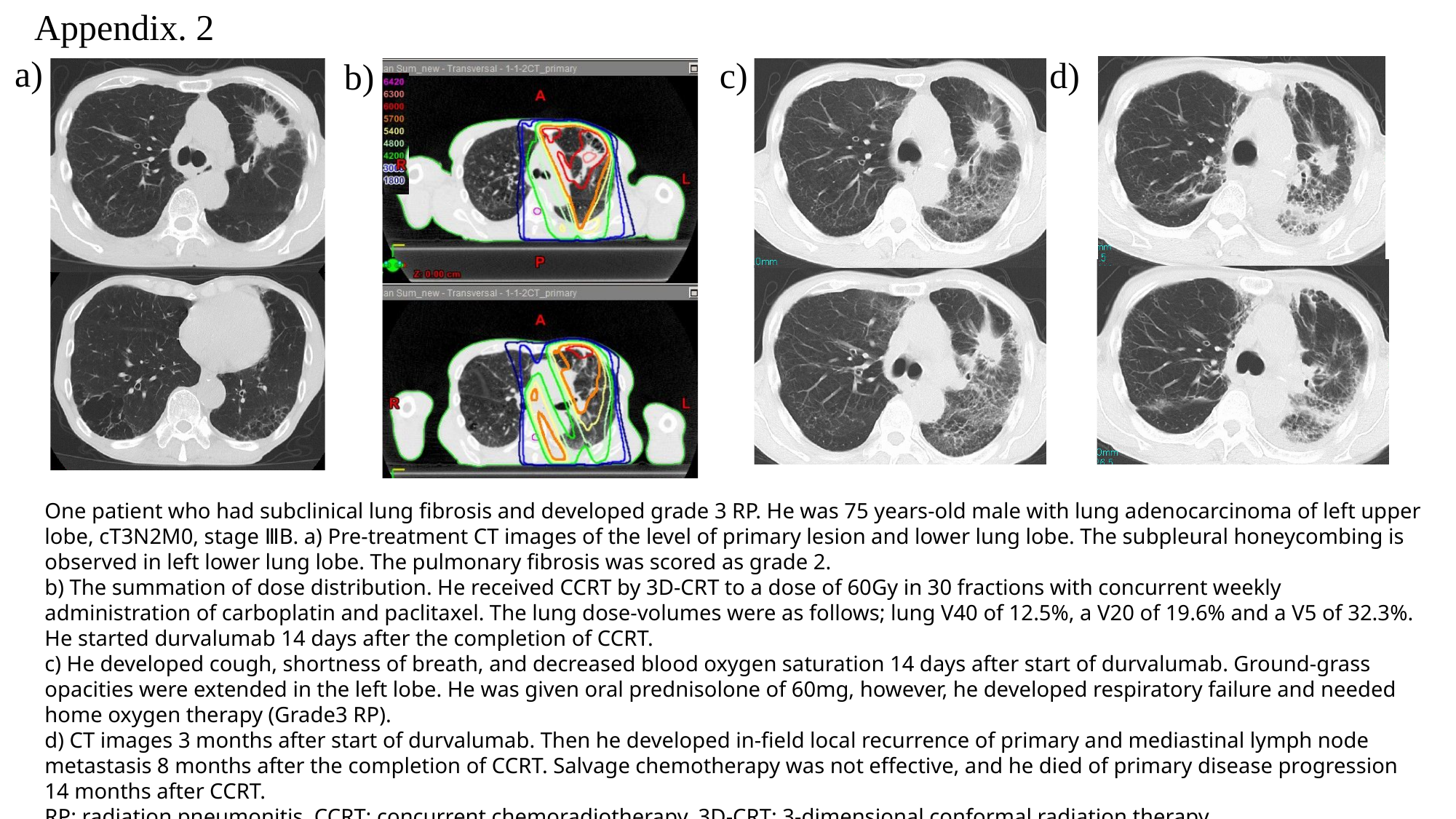

Appendix. 2
a)
c)
d)
b)
One patient who had subclinical lung fibrosis and developed grade 3 RP. He was 75 years-old male with lung adenocarcinoma of left upper lobe, cT3N2M0, stage ⅢB. a) Pre-treatment CT images of the level of primary lesion and lower lung lobe. The subpleural honeycombing is observed in left lower lung lobe. The pulmonary fibrosis was scored as grade 2.
b) The summation of dose distribution. He received CCRT by 3D-CRT to a dose of 60Gy in 30 fractions with concurrent weekly administration of carboplatin and paclitaxel. The lung dose-volumes were as follows; lung V40 of 12.5%, a V20 of 19.6% and a V5 of 32.3%. He started durvalumab 14 days after the completion of CCRT.
c) He developed cough, shortness of breath, and decreased blood oxygen saturation 14 days after start of durvalumab. Ground-grass opacities were extended in the left lobe. He was given oral prednisolone of 60mg, however, he developed respiratory failure and needed home oxygen therapy (Grade3 RP).
d) CT images 3 months after start of durvalumab. Then he developed in-field local recurrence of primary and mediastinal lymph node metastasis 8 months after the completion of CCRT. Salvage chemotherapy was not effective, and he died of primary disease progression 14 months after CCRT.
RP: radiation pneumonitis, CCRT: concurrent chemoradiotherapy, 3D-CRT: 3-dimensional conformal radiation therapy.
